# Supplementary material for: Content-rich biological network constructed by mining PubMed abstracts
Source: BMC Bioinformatics. 2004 Oct 8;5:147. doi: 10.1186/1471-2105-5-147 (PMC528731; doi:10.1186/1471-2105-5-147)
Supplement: Additional File 2 — The original results of the above study (non-essential files are deleted to keep the file size under the limit set by BMC bioinformatics). [file 1471-2105-5-147-S2.bz2 › chilibotAdditionalFile2/dip50/620ID8598903E11005/html/F7_F3.html]

 


 **F7** and **F3** 
  
Found 158 abstracts in PubMed, retrieved 50.  
 

 What does Google say? 
 PDF only 
| .edu only 

---

**Interactive relationship** (e.g. stimulation, inhibition, etc)

**Neutral relationship**- ...  **F3**  and  **F7**  were able to bind to all Ole e 1 variants.  Ref: 12213332 Mol Immunol, 2002
- Tissue factor  [ **F3** ]  TF is the cellular receptor and cofactor for blood coagulation factor VII  [ **F7** ]  FVII.  Ref: 12823950 Trends Pharmacol Sci, 2003
- Tissue factor  [ **F3** ]  TF is a transmembrane glycoprotein that serves as an essential cofactor for plasma coagulation factor VII  [ **F7** ] .  Ref: 10744153 Thromb Haemost, 2000
- BACKGROUND Initiation of the coagulation serine protease cascade in mammalian cells is mediated by tissue factor  [ **F3** ]  TF, which is a cell surface receptor and cofactor for coagulation factor VII  [ **F7** ]  FVII and its activated form FVII FVIIa.  Ref: 11876468 Mol Biotechnol, 2002
- Tissue factor  [ **F3** ]  TF is the main initiator of the coagulation system and functions as a cell surface receptor for coagulation factor VII  [ **F7** ]  FVII.  Ref: 11841337 Biochemistry Mosc , 2002
- One candidate protein to cause coronary NR is tissue factor  [ **F3** ]  TF, which is abundant in atherosclerotic plaque and a cofactor for activated plasma coagulation factor VII  [ **F7** ] .  Ref: 11929768 Blood, 2002
- The starting point of blood coagulation in vivo is the formation of a complex between tissue factor  [ **F3** ]  TF, which is exposed following vascular disease or trauma, and activated blood coagulation factor VII  [ **F7** ]  FVIIa.  Ref: 10850581 Blood Coagul Fibrinolysis, 2000
- Recombinant activated coagulation factor VII  [ **F7** ]  rFVIIa NovoSeven was developed for treatment of bleeding in hemophilia patients with inhibitors antibodies against factors VIII or IX. rFVIIa initiates the coagulation cascade by binding to tissue factor  [ **F3** ]  at the site of injury and causes the formation of sufficient amounts of thrombin to trigger coagulation.  Ref: 11547359 Semin Thromb Hemost, 2001

**Non-interactive relationship** (e.g. studied together, co-existance, homology, etc.)

- The non primary motor cortex Brodmann s area 6 lies further rostrally and can be subdivided into three groups of areas the supplementary motor areas SMA proper area  **F3**  and pre SMA area F6 on the mesial cortical surface, the dorsolateral premotor cortex areas F2 and  **F7**  on the dorsolateral convexity, and the ventrolateral premotor cortex areas F4 and F5 on the ventrolateral convexity.  Ref: 11131014 Anat Embryol Berl , 2000
- Power values of particular frequency bands delta, theta, alpha 1, alpha 2, beta 1, beta 2 were observed on the regions Fp I, Fp2,  **F3** , F4,  **F7** , F8, C3, C4, T3, T4, T5, T6, P3, P4, O1 and O2.  Ref: 11165355 Int J Psychophysiol, 2001
- Although the binding of human IgE to Ole e 1 was not affected by heat treatment, it was shown to be strongly dependent on the integrity of the disulfide bridges and was partially inhibited by  **F3**   **F7**  IgG antibodies, their individual values ranging from 12 to 31% and reaching 53% with their mixture.  Ref: 12213332 Mol Immunol, 2002
- To solve the urgent problem, five fungal species F2,  **F3** , F4,  **F7** , F15 which can effectively degrade phenolic acid were screened to degrade the toxic material in successive plantation site and to improve the soil bioactivity.  Ref: 11758409 Ying Yong Sheng Tai Xue Bao, 2001
- Contingent negative variation CNV was recorded from electrodes  **F7** ,  **F3** , Fz, F4, F8, T7, C3, Cz, C4, T8, P7, P3, Pz, P4 and P8 in 19 young mean age 23 years and 15 elderly mean age 66 years healthy right handed subjects, using a S2 choice paradigm.  Ref: 10696812 Neurosci Lett, 2000
- It was observed that F1, F2,  **F3**  and F4 were highly antigenic, F6 and  **F7**  were moderately antigenic and F5 and F8 were poorly antigenic.  Ref: 10729642 Vet Parasitol, 2000
- Increased SCD was observed at left frontotemporal FT3, bilateral temporoparietal TP3 and TP4 and prefrontal locations F8 F4 and  **F7**   **F3** , suggesting novelty P3 generators located in the left auditory cortex, and bilaterally in temporoparietal and prefrontal association regions.  Ref: 12706218 Brain Res Cogn Brain Res, 2003
- A negative going SCP starting at ca.1.5 2.0 s preceding the onset of the self paced jaw opening and jaw closing movements was found at all the recorded sites of Fz, Cz, Pz,  **F3** , F4,  **F7** , F8, C3, C4, T3 and T4.  Ref: 11186211 J Med Dent Sci, 1998
- MRCPs were recorded in 20 right handed healthy young subjects with electrodes placed at Fp1, Fp2,  **F7** ,  **F3** , F4, F8, T7, C5, C3, C1, C2, C4, C6, T8, P7, P3, P4, P8, O1 and O2.  Ref: 11856630 Clin Neurophysiol, 2002
- In such women the arterial intima can express tissue factor  [ **F3** ] , and changes in coagulation factor VII  [ **F7** ]  factor VII and tissue factor  [ **F3** ]  pathway inhibitor TFPI may be deleterious.  Ref: 12456630 Hum Reprod, 2002
- Effects of different progestin regimens in hormone replacement therapy on blood coagulation factor VII  [ **F7** ]  and tissue factor  [ **F3** ]  pathway inhibitor.  Ref: 12456630 Hum Reprod, 2002
- Coherence scores were calculated for homologous interhemispheric electrode pairs Fp1 Fp2,  **F3**  F4,  **F7**  F8, C3 C4, P3 P4, O1 O2, T3 T4, T5 T6 and for left and right intrahemispheric pairs for delta, theta, alpha, beta1, and beta2 frequencies.  Ref: 10590819 Brain Cogn, 1999
- The other fractions showed anti inflammatory activity in the following order F6> F2> F5> F4> F8>  **F3** >  **F7** > CE.  Ref: 11059504 J Altern Complement Med, 2000
- After varimax rotation, we obtained seven independent factors F1 Disinhibition instability, F2 Paranoia hostility,  **F3**  Deficit, F4 Grandiosity psychosis, F5 Elation euphoria, F6 Depression, and  **F7**  Hyper sexuality.  Ref: 12507733 J Affect Disord, 2003
- Among these, tissue factor  [ **F3** ]  and its ligand coagulation factor VII  [ **F7** ] , thrombin and its protease activated receptors, and type II trans membrane serine proteases seem to play a crucial role.  Ref: 12067200 Clin Exp Metastasis, 2002
- A total of eight major fractions F1, F2,  **F3** , F4, F5, F6,  **F7** , and F8 were separated from the whole homogenate of G. crumenifer, and each of these fractions was tested for their antigenicity by ELISA against rabbit hyperimmune sera.  Ref: 10729642 Vet Parasitol, 2000
- The international normalized ratio INR, developed by the World Health Organization in the early 1980s, is designed to eliminate problems in oral anticoagulant therapy caused by variability in the sensitivity of different commercial sources and different lots of thromboplastin  [ **F3** ]  to blood coagulation factor VII  [ **F7** ] .  Ref: 10797608 J Clin Lab Anal, 2000
- Tissue factor  [ **F3** ]  TF, a transmembrane receptor for the serine protease coagulation factor VII  [ **F7** ]  a FVIIa, is the main initiator of the coagulation cascade.  Ref: 11973337 J Biol Chem, 2002
- those addressed to peri arcuate zone of F2 arise mainly from F5 and, to a lesser extent, from  **F7** , F4, dorsal CMAd and ventral CMAv cingulate motor areas, pre supplementary F6 and supplementary  **F3**  motor areas.  Ref: 11375913 Cereb Cortex, 2001
- Asymmetry scores were computed log right log left for F4  **F3** , Fp2 Fp1, F8  **F7** , C4 C3, T4 T3, T6 T5, P4 P3, and O2 O1 for aggregated eyes open and eyes closed baselines.  Ref: 11252841 J Gend Specif Med< MedlineTA>J Gend Specif Med, 2001
- A frequent human coagulation Factor VII  [ **F7** ]  mutation A294V, c152 in loop 140s affects the interaction with activators, tissue factor  [ **F3** ]  and substrates.  Ref: 11931672 Biochem J, 2002
- The EEG frequency spectrum was subdivided into 1 Hz wide bands and was recorded from electrodes F1, Fpz, Fp2,  **F7** ,  **F3** , Fz, F4, F8, T3, C3, Cz, C4, T4, T5, P3, Pz, P4, T6, O1, Oz and O2.  Ref: 11168778 J Intellect Disabil Res, 2001
- Log transformed values of two qEEG bands, i.e. 2 6 and 6.5 12 Hz, were averaged between adjacent channels frontal  **F3**  and  **F7** , F4 and F8.  Ref: 12207147 Neuropsychobiology, 2002
- Tissue factor  [ **F3** ]  TF, a cell surface associated cofactor and activator of coagulation factor VII  [ **F7** ] , has been implicated in the local and systemic activation of coagulation associated with sepsis.  Ref: 12658585 Vet Clin Pathol< MedlineTA>Vet Clin Pathol, 2002
- and monopolar relative power in theta band for Fp1, Fp2,  **F7** ,  **F3** , Fz, F4 and F8.  Ref: 10973109 Arq Neuropsiquiatr, 2000
- Power characteristics of the EEG theta and alpha rhythms were studied in a human in neutral state and during a conditioned negative emotional reaction Fp1, Fp2,  **F3** , F4, C3, C4, P3, P4, O1, O2,  **F7** , F8, T3, T4, T5, and T6 derivations.  Ref: 11764515 Zh Vyssh Nerv Deiat Im I P Pavlova< MedlineTA>Zh Vyssh Nerv Deiat Im I P Pavlova, 2000
- We found that only fractions  **F3**  and  **F7**  inhibit the tested enzyme.  Ref: 12490243 Fitoterapia, 2002
- RESULTS AND CONCLUSIONS Greater values of delta relative amplitude were found in the attention deficit hyperactivity disorder group in their basal EEG at the  **F7** , F8, FP1, FP2,  **F3** , F4 and C3 sites.  Ref: 11299475 Rev Neurol< MedlineTA>Rev Neurol, 2002
- Fluorinated analogues of propranolol, namely trifluoroethyl propranolol  **F3** , pentafluoropropyl propranolol F5, and heptafluorobutyl propranolol  **F7** , were found to induce reactive oxygen metabolite ROM production in human neutrophils in a dose dependent manner.  Ref: 11239494 Biochem Pharmacol, 2001
- Local frontal projections to F2 pre CD mostly stem from F4, and, to a lesser extent, from  **F7**  and  **F3** , and CMAd.  Ref: 11375913 Cereb Cortex, 2001
